# Novelty, attention, and challenges for developmental psychology

**DOI:** 10.3389/fpsyg.2013.00491

**Published:** 2013-08-01

**Authors:** Emily Mather

**Affiliations:** Department of Psychology, University of HullHull, UK

## Introduction

In this brief essay, I seek to demonstrate the significance of exploratory behavior for understanding cognitive development. Historically, organisms were thought to act solely in the service of achieving biologically significant goals, such as satisfying thirst, hunger, and reproductive drives. However, it became apparent that both animals and humans engage in behavior where the adaptive goal is unclear (see Hunt, 1963, 1965). With no obvious external target, this activity is best described as being intrinsically motivated, and often directed toward the unknown and the unexpected (Kagan, 2002). Hence *novelty*, the discrepancy between what is known and what is discovered, can elicit activity and exploration of the environment.

What is the relevance to developmental process? Attention to novelty plays a seemingly simple role in learning and development, directing the senses toward what is as yet unknown. Yet, research shows that patterns of attention to novelty are not straightforward, particularly during infancy. There is considerable evidence that attention is sometimes biased toward *familiarity*, rather than novelty. Unlike our understanding of novelty preference, we struggle to understand when and why familiarity preferences occur. Below I briefly review this area of research and illustrate how this basic aspect of learning continues to puzzle developmental psychologists.

## From animals to infants?

The habituation mechanism, which directs attention to novelty, has been widely-studied across the animal kingdom (Sokolov, 1963; Thompson and Spencer, 1966). Thorpe (1963) defined habituation as “the relatively permanent waning of a response as a result of repeated stimulation … ” (p. 61). An important feature of habituation is that an organism's responding will recover to the presentation of a different stimulus—an effect known as dishabituation (Thompson and Spencer, 1966). Hence, habituation is stimulus–specific and attention will recover to novel stimuli. A seminal study by Fantz (1964) demonstrated that infants' visual attention to a familiar, repeated image will decrease relative to their attention to a novel image. Other early studies of infant habituation also reveal infants' interest in novel stimuli (see Cohen and Gelber, 1975, for a review). There is evidence that even newborns will habituate and direct their attention to novelty (Friedman, 1972; Slater et al., 1982, 1984). Many infancy researchers are interested in the use of novelty preferences as a methodological tool. Habituation–dishabituation procedures are used to demonstrate infants' discrimination of stimuli, and seemingly precocious cognitive abilities (e.g., Baillargeon, 1987; Spelke et al., 1992; but see Schilling, 2000, for a counterargument).

Infants' interest in novelty is consistent with theories of habituation accounting for both human and animal responding. However, there is substantial evidence that infants do not *always* prefer a novel stimulus—sometimes they prefer to attend to a familiar stimulus. In Rose et al. (1982) groups of 3.5- and 6.5-month-olds were exposed to a visual stimulus for different durations. This familiar stimulus was then paired with a novel stimulus. Infants at both ages displayed familiarity preferences after shorter exposures, and novelty preferences after longer exposures. Similarly, Hunter et al. (1983) presented 8- and 12-month-olds with a set of toys, and tested their preference for the familiar vs. novel toys after differing amounts of familiarization. The infants preferred the familiar toys after a shorter familiarization period, and the novel toys after a longer familiarization period. In these studies, familiarization time was manipulated between groups of infants. Roder et al. (2000) provide a within-infants demonstration that 4.5-month-olds preferences shift from familiarity to novelty as a function of familiarization time.

While familiarity and novelty preferences have largely been investigated for the visual modality, there is also evidence that infants display these preferences for auditory stimuli (e.g., Colombo and Bundy, 1983; Spence, 1996). More recent research has found that infants' preferences will also “reverse” as their memory for a familiarized stimulus decays over time. In Bahrick and Pickens (1995), 3-month-olds displayed a novelty preference after a 1 min retention interval, and a familiarity preference after a 1 month interval (see also Spence, 1996; Bahrick et al., 1997; Courage and Howe, 2001). Familiarity preferences do not just occur to repetitions of a specific stimulus. Infants who have categorized a set of stimuli will sometimes attend more to a novel stimulus from the same category, rather than a novel stimulus drawn from a novel category (e.g., Gomez and Gerken, 1999; Fiser and Aslin, 2002; Gómez and Maye, 2005; Mather and Plunkett, 2011). Hunter and Ames (1988) provide a descriptive model of infants' familiarity and novelty preferences. The main factor is familiarization time—with briefer exposures, the infant attends more to a familiar stimulus, but with longer exposures, their attention turns to novelty. How quickly an infant makes this “familiarity-to-novelty shift” will depend on their processing speed and the complexity of the stimuli. Hence, familiarity preferences are more likely for younger infants (slower processors), and for more complex stimuli.

Familiarity preferences are not consistent with the habituation process, where attention simply declines with repeated exposure to a stimulus (Thompson and Spencer, 1966). Some computational models of infant attention have tentatively linked familiarity preferences with the process of *sensitization* (Sirois and Mareschal, 2004; Schoner and Thelen, 2006). Sensitization occurs when the presentation of a stimulus leads to heightened behavioral responding. Importantly, if a stimulus is repeated, it can have the effect of sensitizing itself. Under dual-process theory (Groves and Thompson, 1970), habituation and sensitization are separate, opposing processes which interact to determine responding. Sensitization is related to stimuli intensity, and decays quickly. If sensitization is initially stronger than habituation, there will be an early increase in responding to a repeated stimulus, followed by a decrease. This pattern of response to a repeated stimulus is similar to the familiarity–novelty shift sometimes evidenced by infants. However, in contrast to habituation, sensitization will generalize to a wide range of stimuli (see Domjan, 1998, for examples). Hence, while sensitization can occur to a repeated stimulus, it would also generalize to other stimuli if they were also present. This means that sensitization cannot account for the stimulus specificity of familiarity and novelty preferences (see also Turk-Browne et al., 2008, for a related argument).

## The old or the new?

An alternative theoretical perspective could account for the existence of familiarity preferences. Since the 1950's, a variety of arguments have been made that both adults and infants prefer stimuli which provide an optimal level of novelty or information (Dember and Earl, 1957; Berlyne, 1960; McCall and McGhee, 1977). The optimum is defined by a “moderate” discrepancy between a stimulus and an observer's representation of that stimulus. Hence, the more discrepant a stimulus is from the observer's state of knowledge, the more novel it is to the observer. Relatedly, stimulus complexity influences the amount of learning required to reduce this discrepancy. Any stimulus which is more or less discrepant than the optimum is of less interest to the observer. The familiarity-to-novelty shift displayed by infants is consistent with optimal-level theory. It is possible for a familiar stimulus to be favored over a novel stimulus, because the familiar stimulus could initially be closer to the optimum. Further processing of the familiar stimulus will result in a shift away from the optimum, and a novel stimulus will be preferred (see Hunter and Ames, 1988, for an elaboration).

A problem with obtaining evidence of the familiarity-to-novelty shift is that there is a temporally limited window for observing a familiarity preference. At a certain point, attention will shift toward novelty, thus a successful experimental design must be sensitive to this shift. Different infants will also process information at different rates, meaning that individual preferences can be obscured by group data (see Roder et al., 2000). Unfortunately, optimal-level theory does not provide a remedy for these methodological issues. The key variables involved—stimulus complexity, processing speed, and the optimal level of novelty—are usually unknown quantities. This makes it difficult to predict the occurrence of familiarity preferences, and to test the assumptions of the theory (see Thomas, 1971). A lack of familiarity preference could be due to the stimuli not being sufficiently complex, or an infant rapidly processing the stimuli. Therefore, while the existence of familiarity preferences is consistent with optimal-level theory, the theory itself perhaps does little more than assert that we seek out moderately novel stimuli.

One approach to dealing with the shortcomings of optimal-level theory has been to develop more computationally explicit models of familiarity and novelty preferences (Sirois and Mareschal, 2004; Schoner and Thelen, 2006; see also Perone et al., 2011) and to mathematically formalize the information content of a stimulus (e.g., Kidd et al., 2012). These recent advances offer an improved level of theoretical precision over past formulations of optimal-level theory. Nonetheless, these models incorporate some of the basic assumptions of optimal-level theory, and may also retain the difficulties of predicting the familiarity-to-novelty shift. Our ability to understand exactly when infants will seek out familiarity or novelty is likely to require a deeper understanding of *why* there is an optimum in the first place. Development requires a balance of familiarization with regularities in the environment (Gibson, 1969) and shifting attention to what is new and unknown so as to create new cognitive structures (Piaget, 1936/1952). Therefore, rather than just focusing on preferences for individual stimuli, one useful approach might be to explore the more global consequences for the abstraction and development of knowledge.

## Order and timing: the cycle of cognitive development

The familiarity-to-novelty shift causes infants to process stimuli in a particular sequence. That is, with all other factors held constant, infants' will explore different stimuli in a systematic fashion, based on their prior experience and learning. Beyond the laboratory, how do these preferences shape patterns of learning across the vast multitude of items and events in the real world, across multiple timescales? Computational models and experimental data demonstrate how the pattern of input can influence the trajectory and success of learning. In Elman (1993), recurrent neural networks were more successful at acquiring grammatical categories if they began by only learning about a subset of the total sentences available, rather than learning about all sentences together (see also Plunkett and Marchman, 1991, 1993). Other research suggests that order effects may also occur in infant categorization (Sandhofer and Doumas, 2008; Mather and Plunkett, 2011). What is particularly intriguing is that in some cases, exposure to an initially restricted stimulus set supports learning (Elman, 1993), whereas in other cases, reduced variability hinders learning (see Mather and Plunkett, 2011). These findings hint at a global effect of optimal preferences on successful learning.

Currently, we understand little about the role that familiarity and novelty preferences might play in driving successful patterns of learning. However, if we can better understand the effects of these preferences on cognitive development, then we might make sense of the underlying cause of optimal preferences. Conversely, our explanations of cognitive development would also benefit from understand the impact of exploratory behavior on learning. Much current developmental research is concerned with specifying the mechanisms of learning, without considering how and why attention prioritizes certain stimuli for learning. Cognitive development needs to be understood as a cyclical process, where attention influences learning, and learning guides attention. If, as Piaget (1936/1952) argued, the child is *actively* engaged in the construction of their own knowledge, then exploratory behavior needs to be placed at the heart of cognitive development.

## Conclusions

A hallmark of human behavior is that we seek to explore and understand our environments, even in the absence of biological or externally specified goals. Our interest in what is new and unknown is evident from birth. However, we do not yet have a clear understanding of the mechanisms which determine whether a child attends to familiarity or novelty. The function of optimal preferences may need to be interpreted in the context of broader developmental changes. Both the processes of cognitive growth and exploratory behavior can be better understood by considering their interdependence.
